# The Effect of Lutein on Eye and Extra-Eye Health

**DOI:** 10.3390/nu10091321

**Published:** 2018-09-18

**Authors:** Silvio Buscemi, Davide Corleo, Francesco Di Pace, Maria Letizia Petroni, Angela Satriano, Giulio Marchesini

**Affiliations:** 1 Dipartimento Biomedico di Medicina Interna e Specialistica (DIBIMIS), University of Palermo, Unit of Endocrinologia, Malattie del Ricambio e Della Nutrizione, Policlinico University Hospital, 90127 Palermo, Italy; davidecorleo@gmail.com; 2 Dipartimento di Biomedicina Sperimentale e Neuroscienze Cliniche (BIONEC), University of Palermo, Unit of Oculistica, Policlinico University Hospital, 90127 Palermo, Italy; francesco.dipace@unipa.it; 3 Dipartimento di Scienze Mediche e Chirurgiche, University of Bologna, SSD di Malattie del Metabolismo e Dietetica Clinica, S. Orsola-Malpighi University Hospital, 40135 Bologna, Italy; marialetizia.petroni@gmail.com (M.L.P.); giulio.marchesini@unibo.it (G.M.); 4 IRCCS Policlinico San Donato, 20097 San Donato Milanese, Italy; angela.satriano@gmail.com

**Keywords:** lutein, carotenoids, antioxidants, eye health, age-related macular degeneration, cataract, cardiovascular health, cognitive performances, cancer

## Abstract

Lutein is a carotenoid with reported anti-inflammatory properties. A large body of evidence shows that lutein has several beneficial effects, especially on eye health. In particular, lutein is known to improve or even prevent age-related macular disease which is the leading cause of blindness and vision impairment. Furthermore, many studies have reported that lutein may also have positive effects in different clinical conditions, thus ameliorating cognitive function, decreasing the risk of cancer, and improving measures of cardiovascular health. At present, the available data have been obtained from both observational studies investigating lutein intake with food, and a few intervention trials assessing the efficacy of lutein supplementation. In general, sustained lutein consumption, either through diet or supplementation, may contribute to reducing the burden of several chronic diseases. However, there are also conflicting data concerning lutein efficacy in inducing favorable effects on human health and there are no univocal data concerning the most appropriate dosage for daily lutein supplementation. Therefore, based on the most recent findings, this review will focus on lutein properties, dietary sources, usual intake, efficacy in human health, and toxicity.

## 1. Introduction

A large body of evidence suggests that a diet rich in antioxidants, which have an anti-inflammatory role [1,2], may contribute to reducing the burden of chronic diseases [3]. Carotenoids are nutrients widely distributed in foods, especially in fruit and vegetables [4], and appear to have antioxidant properties [5,6]. In recent decades, there has been increasing interest in their effects on health; a high dietary intake of carotenoids has been associated with beneficial effects in several systemic diseases [7] and in eye disorders, with protection of the retina from phototoxic light damage [8]. Most studies have focused on lutein (L), a carotenoid with a strong antioxidant effect in vitro [9] that has been associated with a reduced risk of age-related diseases [10]. Lutein is a xanthophyll, i.e., an oxygenated carotenoid that all mammalians, humans included, derive from their diet because they are unable to synthesize carotenoids [11]. Several studies have shown that high L intake, either through diet or as nutritional supplement, has beneficial effects on eye diseases, preventing or even improving both age-related macular degeneration (AMD) [12,13,14,15,16] and cataract [17,18,19,20,21]. However, conflicting data had been reported concerning L efficacy [22,23,24,25,26,27,28,29,30], and in 2006, it was claimed that no compelling evidence had been provided concerning the supposed beneficial effect of L on human health [31]. Furthermore, no univocal data concerning the appropriate dosage for possible L supplementation had been found [32,33,34,35,36,37,38]. More recently, a number of studies have suggested that L may indeed have favorable effects via anti-inflammatory activity [39], improving cognitive functions [40], and decreasing the risk of cancer [41], cardiovascular diseases [39] and other systemic conditions [42,43,44]. Overall, it seems that the amount of L intake, including by supplementation, may partly counter inflammatory processes and favor human health, but inconsistencies still remain.

We reviewed the literature on the evidence for the health effects of L, including its usual intake with different diets, adequate doses, and safety of supplementation, with specific reference to eye diseases.

## 2. Characteristics of Lutein

### 2.1. Structure and Distribution

**Figure 1:**
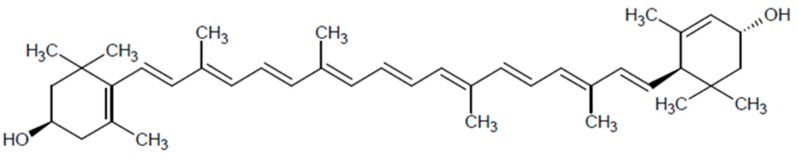
Structure of lutein.

The structure of L is similar to that of other carotenoids, with a skeleton made up of 40 carbon atoms, organized into eight isoprene units [45], as shown in Figure 1. However, an important chemical difference with functional implications is the presence of two oxygen atoms inside the structure [46], thus making L a polar carotenoid which is classified as a xanthophyll, namely an oxygenated carotenoid. With zeaxanthin (Z), another xanthophyll, L is the main carotenoid in the human macula [45], so that the two compounds are mostly referred to as macular pigments (MP). Lutein is found mainly in the inner plexiform layer and in Henle’s fiber layer [47] but it can also be found in Müller cells [48]. The presence of L has also been demonstrated in peripheral regions of the fovea [49], but its content decreases in the central region where Z is prevalent [49] by a 2:1 ratio [50]. Interestingly, the content of carotenoids diminishes significantly, by a factor of 100, moving away from the macula [51]. In infants, the macular levels of L are higher than those of Z, probably due to differences in transport mechanisms that are not yet completely developed [49]. On the other hand, Bernstein et al. reported that uveal structures account for about 50% of total eye carotenoids and 30% of total eye L [52]; this is the basis for the possible beneficial effects that L may have in the ciliary body and in the iris. Sato et al. suggested that L uptake in the retina may be mediated by a specific transporter, namely scavenger class B type 1 [53], thus explaining the massive build-up of L in the eye. Finally, L is significantly detectable not only in the eye, but also in brain, where it represents the main carotenoid, especially in infants [54] and in the elderly [55].

### 2.2. Absorption and Metabolism

As reported above, mammalians are not able to synthesize carotenoids which therefore need to be introduced with food [11]. Once ingested, L is absorbed by the mucosa of the small bowel and bound to chylomicrons; then, it is secreted into lymph and reaches the liver [56]. In hepatocytes, L is incorporated into lipoproteins that are distributed to peripheral tissues [57,58], particularly the retina, where the highest concentrations have been demonstrated [59]. L is fat-soluble [60], hence the dietary content of lipids mediates L absorption through its incorporation into micelles [61], and several dietary factors may compete. A diet rich in fiber has been reported to reduce carotenoid serum levels [62,63], also affecting L absorption, whereas the presence of other carotenoids in diet might interfere with L assimilation, probably via a competitive mechanism [64]. The content of iron and zinc as well as protein deficiency may affect L absorption [65]; conversely, the presence of mono- and di-glycerides is likely to positively regulate L absorption, as suggested by significantly increased L plasma levels [66]. Finally, extra-dietary factors may reduce L bioavailability. Orlistat, a drug that inhibits lipase activity, has proven to decrease L absorption [67] to an extent similar to that of impaired activity of pancreatic enzymes [68], as in the case of in vitro measurements relative to patients with cystic fibrosis [69] or, to a lesser extent, with smoking [70] and alcohol consumption [71].

### 2.3. Mechanisms of Action

Both animal and in vitro studies demonstrated that some carotenoids are compounds with antioxidant activity [5,6]. Lutein has been demonstrated to exert an extremely potent antioxidant action by quenching singlet oxygen and scavenging free radicals [9,72], although it seems to be less potent than Z [9]. Another protective effect of L consists in the ability of filtering blue light, thus reducing phototoxic damage to photoreceptor cells [73]. Subczynski et al. hypothesized that L properties might be amplified by its localization in the most vulnerable regions of the retina and by the specific orientation in membranes [74]. Notably, several studies have observed that L inhibits both the pro-inflammatory cytokine cascade [39] and the transcription factor nuclear factor-kB (NF-kB) [75,76,77]. There is also compelling evidence that L reduces reactive oxygen species (ROS) production [75,78], the expression of inducible nitric oxide synthase (iNOS) [79] and the activation of the complement system [80]. Through all these mechanism(s), it is quite conceivable that L may exert a pivotal role in regulating immune pathways, modulating inflammatory responses, and reducing oxidative damage.

## 3. Dietary Lutein Intake

Lutein is naturally abundant and available in fruit, cereals, and vegetables, and it is also present in egg yolk [4], as seen in Table 1, where its bioavailability is higher than in any other food [81]. Since L intake depends on vegetable consumption, it may vary according to dietary habits, in a range that has been estimated from 0.67 mg/d to more than 20 mg/d [82]. In individuals consuming a Western-style diet, the average daily L intake has been estimated at 1.7 mg/d [83] while in countries that consume a Mediterranean diet rich in fruit and vegetables it has been reported to be between 1.07 [84] and 2.9 mg/d [85], with large inter-country variability. In a Korean population, the average L intake was estimated at about 3 mg/d [86]. Interestingly, the highest L intake was reported in Pacific countries, where individuals consume a diet extremely rich in fruit and vegetables, reaching the peak of about 25 mg/d in the Fiji Islands [87].

## 4. Lutein and Eye Disturbances

### 4.1. Age-Related Macular Degeneration

Age-related macular degeneration (AMD) is the main cause of vision impairment and blindness in developed countries [89,90]. Low habitual consumption of green leafy vegetables and fruit is one of the risk factors of AMD [91]. To date, many studies have observed positive effects of L in terms of improvement of macular pigment optical density (MPOD) levels [12,14,16,32,34,35,36,37,38,92,93,94,95,96,97,98,99,100], visual acuity (VA) [12,15,33,35,95,101,102] and contrast sensitivity (CS) [12,24,33,36,37,93,95,96,99,100,102]. The original studies that first investigated a possible protective role of L against AMD date back to the early 1990s. In a case-control study including 421 individuals with neovascular AMD and 615 controls, it was found that the odds ratio (OR) of developing AMD was 0.3 when the highest quintile of L serum concentrations was compared with the lowest quintile, hypothesizing a negative relationship between L levels and AMD risk [103]. In the same cohort, the dietary L intake was associated with the risk of AMD, and again an OR of 0.43 was observed comparing the highest vs. the lowest quintile of dietary L intake [10]. Following these observational studies, the efficacy of nutritional supplementation with L was investigated in intervention studies. The Lutein Antioxidant Supplementation Trial (LAST) included 90 individuals with atrophic AMD and demonstrated a significant beneficial effect of L supplementation (10 mg/d), either alone or in combination with other antioxidants, for nearly 1 year [12]; in particular, it was observed that L enhanced MPOD, also improving VA and CS [12]. The Carotenoids and Antioxidants in Age-Related Maculopathy Italian Study (CARMIS) demonstrated that L supplementation (10 mg/d for 1 year) in people with non-advanced AMD improved the dysfunction in the central retina assessed by multifocal electroretinograms, obtaining benefits also in terms of VA and glare sensitivity [13]. According to a more recent study, L supplementation (20 mg/d for 3 months, followed by 10 mg/d for another 3 months) significantly increased MPOD by about 28% compared to placebo in 126 participants with AMD [14]. The Carotenoids with Co-antioxidants in Age-Related Maculopathy (CARMA) study demonstrated that L (12 mg/d for 2 years) improved VA in 433 patients with early AMD [15]. Furthermore, in the Combination of Lutein Effects in the Aging Retina (CLEAR) study, the MPOD of 72 patients with AMD significantly increased after one year of L supplementation (10 mg/d) [16].

The Age-Related Eye Disease Study 2 (AREDS2) is the most important and recent randomized controlled trial (RCT) to have assessed AMD treatment with oral supplementation of vitamins and micronutrients, including L. The original AREDS oral supplementation had already proven its efficacy in reducing the risk of developing advanced AMD [104]. In order to analyze the effect of adding L (10 mg/d) and Z (2 mg/d) to the original formulation [vitamin C (500 mg), vitamin E (400 IU), beta-carotene (15 mg), zinc (80 mg), and copper (2 mg)], the AREDS2 study included more than 4000 individuals at risk of developing late AMD. The trial failed to prove the efficacy of L in reducing the progression to advanced AMD or in improving VA [27]. However, a 26% reduction of risk for late AMD was observed in individuals in the lowest quintile of L dietary intake who also received AREDS2 supplementation [105], especially in subjects with large drusen. In this study other formulations were also investigated. The AREDS formulation + L, with and without beta-carotene, was compared to the original AREDS formulation, because of the possible increased risk of lung cancer associated with beta-carotene consumption, especially in smokers [106,107]; AMD progression was significantly reduced in the L enriched group, suggesting that L might replace beta-carotene, improving treatment safety [105].

Genetic factors are thought to influence the risk of AMD, in particular mutations involving genes encoding complement factor H (CFH). Interestingly, Ho et al. [108] demonstrated that higher antioxidant and L intakes significantly decreased the risk of developing AMD in individuals with CFH genes variants, probably neutralizing the augmented genetic risk. Two recent meta-analyses including L randomized clinical trials (RCTs) demonstrated beneficial effects of L on MPOD [32] and VA [33] development. However, other studies failed to demonstrate that L supplementation was able to improve both VA [14,16,22,23,26,27,32,36,92,93,98,99] and CS [15,26]. In particular, it was also shown that L was ineffective in reducing the risk of developing AMD [29,30] and in slowing progression to late AMD [27,92]. According to the meta-analysis by Chong et al., L was unable to diminish the risk of developing AMD [29]. These differences between the studies might be partly explained by the differing duration of L supplementation, or by differences in clinical characteristics of patients enrolled in the trial protocols. Therefore, though many studies concluded that L may be able to prevent or treating AMD, additional RCTs are needed to elucidate and characterize the therapeutic properties of L. A list of the intervention studies that have assessed the effect of L on visual performance is presented in Table 2.

Similarly, the issue of the most appropriate dosage for L supplementation is unsolved. According to three studies, L supplementation with 20 mg/d was not more effective than the dose of 10 mg/d in improving visual performance [34,35,36]. Similar results were also obtained by a meta-analysis of 5 RCTs [32]. Conversely, other studies were consistent with a linear dose-response pattern for L efficacy [37,38], supported by a meta-analysis of 8 RCTs [33], suggesting that the higher the L intake, the better the outcome. These conflicting results very likely stem from differences in the study population with specific reference to dietary intake. More than 20 years ago Seddon et al. reported that a L intake of at least 6 mg/d was protective against AMD [10] and AREDS2 showed that the population in the lowest quintile of dietary intake (with a median consumption of only 0.7 mg/d per 1000 kcal of energy) had the most positive results from L supplementation [105]. Therefore, in the presence of an insufficient average intake of L of approximately 1.4 mg/d, supplementation of 10 mg/d, as in the AREDS2, might be the most appropriate dosage for chronic L supplementation.

### 4.2. Cataract

Cataract, as well as AMD, is a growing health problem responsible for vision loss [110], due to oxidation of lens structures. Antioxidants, specifically L, might be a safe treatment for this condition, and in-vitro studies have demonstrated that L is able to prevent cataract in bovine cells by inhibiting the proliferation and migration of lens cells [111], as well as to prevent ultraviolet damage in human lens cells [109]. A few observational studies have found a significant correlation between high L plasma concentrations and a low risk for developing cataract [112,113,114,115], and a negative association has also been reported between daily L intake and the risk of cataract, especially nuclear cataract [17,18,19,20,21]. In particular, in over 30,000 participants Brown et al. observed a significant reduction (−19%) in the risk of cataract in the highest quintile of L intake compared with the lowest quintile [20]. Similar results were obtained by both Chasan-Taber et al. [21] and Moeller et al. [19]. Along this line, Olmedilla et al. demonstrated that L supplementation for 2 years (15 mg/d) was effective in improving visual function in subjects with age-related nuclear cataract [116], but these beneficial effects are controversial [117]. Lyle et al. showed that neither high L serum levels had an effect on the incidence of cataract [118], nor was there a correlation between the disease and L intake [119]. Also, the AREDS2 study showed that L treatment was ineffective both in preventing vision loss and in slowing progression towards cataract surgery [120]. However, a meta-analysis reported a significant negative association between L serum levels and risk of nuclear cataract [121] and another meta-analysis of 6 cohort studies including more than 40,000 participants concluded that daily L intake was negatively associated with the risk of developing nuclear cataract in a dose-linear response [122]. Mares-Perlman et al. suggested that these uncertainties could be explained by limitations in the studies and by the different types of cataract [123] and, irrespective of conflicting data, Weikel et al. concluded that L might be useful in cataract treatment [124]. We definitely need more data, and carefully conducted RCTs evidence on this issue consisting of prospective clinical studies that use data obtained from database of patients who had been prescribed lutein to slow the progression of cataract. The possibility to effectively treat this disabling disease with a safe nutritional intervention is a key issue and needs to be clarified.

### 4.3. Other Eye Diseases

The effect of L has also been also investigated for other eye diseases, but the results have generally been unsatisfactory. A recent RCT reported that L supplementation (10 mg/d) for 36 weeks in patients with diabetic retinopathy (DR) improved only CS, without any effect on VA [125]. The Atherosclerosis Risk in Communities (ARIC) study showed that L intake was not associated with DR in spite of the marked antioxidant properties [126].

In retinitis pigmentosa, 24-week L supplementation significantly increased the visual field in one study [127], but other studies [128,129] failed to confirm this finding.

Retinopathy of prematurity (ROP) is an oxidative-based disease [130], and it has been hypothesized that L might be useful in managing this condition thanks to its anti-oxidant properties. A recent study in mice demonstrated that L significantly ameliorated retinal revascularization [131], but in preterm infants L failed to prevent ROP [132]. In this case as well, more research is eagerly awaited.

## 5. Extra-Eye Actions

### 5.1. Cognitive Function

Given the presence of L in the brain, much research has been conducted to investigate the potential of L in slowing down age-related cognitive decline and eventually how to recover damaged cognitive functions. Lutein is considered to have a significant role in preserving neural efficiency. In a cohort of centenarians, Johnson et al. observed that higher circulating L levels were associated with better cognitive performance [40] and similar results were also reported in the general population of older adults [133]. Higher L circulating levels have been correlated with better cognitive ability scores, as measured by the Wechsler scale [134], and it has been proposed that an increased parahippocampal cortex volume may account for these results. Interestingly, Picone et al. observed a positive correlation between L and blood concentrations of activin-A in infants [135], a neuroprotection marker [136,137]. In older women, Johnson et al. demonstrated that L supplementation significantly improved some cognitive functions, namely verbal fluency, memory scores and efficient learning [138]. Recent data also demonstrated that L is effective in improving memory [139] or other measures of cognitive function, such as spatial memory and reasoning ability [140]. By functional magnetic resonance imaging, it has been observed that L may improve cerebral perfusion [141] or neural efficiency in older adults [142] and two studies have demonstrated a relationship between MPOD and some aspects of cognitive function as prospective memory [143] or verbal fluency and processing speed [144]. Furthermore, Kuchan et al. [145] reported that L stimulates the in-vitro differentiation of human stem cells into neural progenitors, a noteworthy effect that might protect against specific types of dementia as Alzheimer’s disease. In mice, L was able to reverse the loss of nigral dopaminergic neurons by reducing oxidative stress and improving mitochondrial dysfunction [146]. Morris et al. prospectively reported that dietary factors associated with L intake slow down age-related cognitive decline [147]. Despite these promising reports, the AREDS2 study did not observe any favorable effect of L on cognitive performance [148]. Given the amount of data supporting a beneficial role of L on brain tissue and the importance of treatments for cognitive disorders, randomized clinical trials on the possible therapeutic role of L should be planned. 

### 5.2. Cardiovascular Health

Atherosclerosis and cardiovascular (CV) health partly depend on the activation of inflammatory cytokines [149]; therefore, nutrients and drugs that favorably influence the cytokine cascade might effectively prevent CV damage. In guinea pigs fed with L-enriched foods a decrease in medium size low density lipoprotein (LDL) was observed [150], associated with reduced aortic cholesterol, reduced oxidized LDL, and a minimally reduced intimal thickening compared to control animals. In mice, L significantly suppressed atherosclerotic plaque formation by increasing peroxisome proliferator-activated receptor-α (PPAR-α) expression [151]. Furthermore, in rats L countered the oxidative stress induced by hyper-homocysteinemia [152], a minor CV risk factor [153,154] and prevented cardiac and renal injury induced by streptozotocin in association with reduced oxidative stress markers [155]. Interestingly, Rafi et al. observed that L significantly decreased the expression of inducible nitric oxide synthase in mice macrophage cells [79], a factor associated with inflammation and atherosclerosis [156].

Many studies in humans have also provided evidence for a beneficial role of L supplementation, lowering the blood concentrations of inflammatory cytokines, while favoring the secretion of anti-inflammatory cytokines [39]. Indeed, L reduced circulating complement factors [157,158], complement system activation [80], and factors potentially harmful to CV health [159]. Other factors influencing atherosclerosis and CV risk, like lipid peroxidation and C-reactive protein serum concentrations, were shown to be reduced following L supplementation [160]. Karppi et al. suggested that L plasma levels were inversely associated with circulating levels of oxidized LDL [161], whereas low circulating levels of L were associated with an increased risk of developing atrial fibrillation [162]. In patients with early atherosclerosis, L supplementation (20 mg/d) for 3 months was associated with a significant reduction in plasma LDL-cholesterol and triglycerides [163]. An RCT conducted on 144 patients with subclinical atherosclerosis demonstrated that L significantly decreased the carotid intima-media thickness [164], which is an established CV risk factor [165]. In mononuclear cells of patients with stable angina or coronary acute syndrome, L negatively regulated the expression of inflammatory cytokines such as interleukin-1b, interleukin-6, and tumor necrosis factor-α [39]. Interestingly, a recent study demonstrated an independent positive association between L plasma levels and telomere length [166], a suggested marker of the aging process and also proposed as a predictor of myocardial infarction [167].

On the contrary, other studies have failed to demonstrate that L is effective in CV prevention. The AREDS2 study found that L supplementation was unable to reduce CV diseases in patients with AMD [168]. Leermakers et al. assessed median L intake in 13-month-old children by administering a food frequency questionnaire to their caregivers but, at 6 years of age, they did not find any improvement in cardiometabolic health [169], suggesting a lack of correlation between L consumption and parameters of CV health. However, a recent meta-analysis by the same authors that included studies enrolling more than 350,000 participants concluded that L consumption is associated with better CV health [170]. Although these data have been obtained mostly from observational studies, they conclude that the risk for coronary disease and stroke is lower in the highest tertile for L intake compared to the lowest. In summary, there is some evidence that L is a CV protective factor, however, definitive data from RCTs are still missing.

### 5.3. Cancer Risk

Cancer is recognized as a multifactorial disease based not only on an uncontrolled cellular growth, but also on immune dysregulation [171] and activation of inflammatory pathways [172]. Indeed, a diet rich in fresh fruit and vegetables is generally considered a protective factor [3]; as such, L might also play a role against cancer. Slattery et al. observed a 17% reduced risk of developing colon cancer in the highest vs. the lowest quintile of daily L consumption [41]; a negative correlation between L intake and risk of pancreatic cancer was also reported [173]; moreover, L appeared to be protective also against breast cancer [174,175,176] and head-and-neck cancer [177]. Interesting results have also come from several meta-analyses investigating the potential of L as an anti-cancer compound. According to Chen et al. L is associated with a decreased risk of non-Hodgkin lymphoma [178], whereas Ge et al. observed a negative association between L intake and esophageal cancer risk [179]. Interestingly, in-vitro studies with L have not reported a cytotoxic effect against normal human colon cells, but a significantly reduced survival rate of colon cancer cells [180]. However, in this case as well, the results are conflicting and findings that fail to support this association have also been reported [181,182,183,184]. At present, there are too many uncertainties about the anti-cancer action of L, but data are sufficient to pursue this research line.

### 5.4. Other Systemic and Metabolic Effects

Lutein might have an important role even in other diseases. In rats fed with high fat diet, Qiu et al. observed that L significantly decreased circulating cholesterol serum levels and hepatic cholesterol and triglycerides [185]. These authors also found that L improved insulin sensitivity by acting on the expression of key factors involved in hepatic signaling, such as sirtuin-1 and PPAR-α. Another experiment carried out on mice demonstrated that L prevented arsenic-induced hepatotoxicity via reduced ROS production and lipid peroxidation [78]. Cao et al. reported a dose-dependent inverse association between non-alcoholic fatty liver disease risk and serum carotenoid levels, including L [43]; in particular, they observed a 44% lower risk in the highest vs. lowest quintile. Accordingly, L might also exert a significant protective action on the liver.

Similarly, L might have a beneficial effect even on lung function, and a high L intake was associated with a significant improvement in forced vital capacity and forced expiratory volume [44]. However, there is probably too little evidence to conclude on this issue [186].

Bone health is of great importance, especially in the elderly as decreased bone mineral density may lead to osteoporosis and fractures [187]. In mice, L significantly stimulated bone formation and inhibited bone reabsorption through its regulatory activity on NF-κB [188]. Similar results were obtained in vitro, where L was effective in increasing bone formation, preventing bone loss, and decreasing the interleukin-1-dependent differentiation of osteoclasts [189]. Observational studies have confirmed the possible beneficial effects of L intake on total hip bone mineral density in men [42], supporting a positive role on bone health [190]. 

Interestingly, an RCT reported that L protected the skin against the damage induced by solar radiation [191]; Palombo et al. reported beneficial effects of both topical and oral L administration on skin elasticity and hydration [192] and similar results were observed by Morganti et al. [193]. However, it seems premature to claim that L exerts a protective effect on skin.

Finally, a few data also exist on a possible role of L in pregnancy, but, Lorenzoni et al. did not find any protective effect of L on oxidative stress in women with gestational diabetes [194]. On the contrary, a case-control study by Cohen et al. suggested that higher plasma L concentrations were associated with low risk of preeclampsia [195]. Despite these findings, data concerning a protective role of carotenoids against as pregnancy diseases as outcomes have been considered inconclusive even by a recent review on the effects of carotenoids during pregnancy [196].

## 6. Lutein Safety and Toxicity

In a well-balanced diet, L intake is sufficient and there is no need for supplementation, but in the presence of inadequate absorption or chronic diseases this possibility needs careful consideration. Several studies have been carried out to establish reasonable upper limits of safety for daily supplementation and to describe possible side effects of chronic L supplementation. To date, no study reported toxicity, either in acute or during chronic L supplementation [197]. Studies performed both in animals [198,199] and in-vitro [199] clearly demonstrated that the use of L is safe as no mutagenic or teratogenic effect was observed. Nevertheless, mice lacking Beta-Carotene Oxygenase 2 exhibited a pathologic carotenoids accumulation and a significant increase in oxidative stress and mitochondrial dysfunction [200] suggesting that an excessive carotenoid supplementation might lead to toxicity under certain conditions. Furthermore, epidemiological studies as well as intervention studies did not observe any toxic effect caused by L [201]. However, according to current evidence, the Joint Expert Committee on Food Additives established an upper safety limit for daily L intake of 2 mg/kg [202], while the European Food Safety Authority (EFSA) was more cautious and indicated a limit of 1 mg/kg [197]. This is consistent with the data obtained by Landrum et al. [203] and Dagnelie et al. [204] who demonstrated that the intake of L is safe up to 30 and 40 mg per day respectively. The EFSA additionally set an upper limit for L-enriched milk for infants, establishing a maximum L supplementation of 250 μg/L [205]. Zheng et al. [206] demonstrated no interactions between L intake and cytochrome P450 enzyme activity; hence it is conceivable that L does not alter the metabolism of other exogenous or endogenous substances. Nevertheless, although L does not seem to be toxic, some side effects have been reported. Indeed, Olmedilla et al. [207] reported that subjects receiving L supplementation of 15 mg/d for 20 weeks developed skin yellowing [carotenodermia], an innocuous but unpleasant side effect. An observational study [208] hypothesized that L might be associated with an increased risk of lung cancer, especially among smokers. In particular, an association was observed between chronic intake of supplements also containing L and an increased risk of lung cancer, mainly non-small cell lung cancer [208]. However, an accurate survey performed by the EFSA concluded that data were insufficient to consider that L supplementation is associated with such negative events [197]. Similarly, the AREDS2 intervention study did not observe any increased incidence of lung cancer with L supplementation suggesting that a health warning was unnecessary. A recent case report described the occurrence of crystalline maculopathy in an old woman on L supplementation and this potential side effect reversed after L intake discontinuation [209]. However, the absence of similar data throughout recent decades makes also this association unlikely. Therefore, based on available data, it is reasonable to conclude that chronic L supplementation at the recommended dose of 10 mg/d, as in the AREDS2 study, is safe and not toxic.

## 7. Conclusions

Lutein qualifies as a powerful antioxidant and many studies support its favorable effects on eye health. Also, L has beneficial effects on other tissues, especially the brain, where it was associated with improved cognitive performance. Thus, not only high L intake with a diet rich in fruit and vegetables, but also its supplementation might be encouraged, particularly in the elderly and in individuals at high risk of different clinical conditions. However, there are still conflicting data that need to be elucidated by randomized clinical trials with large cohorts of general population. Furthermore, most of the results available at present were obtained from clinical trials lasting less than 1 year. Therefore, this time span is probably not sufficient to show significant favorable effects; therefore, studies with a longer duration are needed to better elucidate the possible favorable role of L on human health.

## Figures and Tables

**Table 1:** Lutein and zeaxanthin content of some fresh foods (mean serving) ^a^.

Food	L + Z Content (mg/100 g of Food)		L + Z Content (mg/Household)	
	Raw	Cooked	Raw	Cooked
Paprika	18.94		0.43 ^t^	
Sweet potato leaves	14.72	11.45	5.15 ^c^	7.32 ^c^
Dandelion	13.61	9.16	7.48 ^c^	9.61 ^c^
Pepper	13.16		0.23 ^t^	
Turnip greens	12.83	8.44	7.05 ^c^	12.15 ^c^
Cress	12.50	8.40	6.25 ^c^	11.34 ^c^
Spinach	12.20	11.31	3.66 ^c^	20.35 ^c^
Chard	11.00	11.02	3.96 ^c^	19.28 ^c^
Chicory	10.30		2.99 ^c^	
Radicchio	8.83		3.53 ^c^	
Kale	6.26	4.98	1.32 ^c^	5.88 ^c^
Basil	5.65		0.14 ^l^	
Parsley	5.56		3.34 ^c^	
Collards	4.32	6.2	1.56 ^c^	11.77 ^c^
Mustard greens	3.73	10.4	2.09 ^c^	14.56 ^c^
Arugula	3.56			
Peas	2.48	2.59	3.59 ^c^	4.15 ^c^
Lettuce	2.31		1.09 ^c^	
Squash	2.13	2.25	2.40 ^c^	4.05 ^c^
Egg yolk	1.09		0.19 ^e^	

^a^ [88]; L, lutein; Z, zeaxanthin; ^c^ cup; ^e^ 1 large egg; ^l^ 5 leaves; ^t^ 1 teaspoon; L.

**Table 2:** Intervention studies on the effects of lutein on visual performance.

Study (year)	Design (Number of Participants)	Intervention and Lutein Supplementation	Effects
AREDS2 (2013) [27]	RCT, participants with bilateral drusen or AMD in 1 eye (4176); 4 groups: G1 (1007); G2 (1038); G3 (1062); G4 (1069)	G1: AREDS formulation; G2: AREDS + L 10 mg + Z 2 mg; G3: AREDS + DHA 350 mg + EPA 650 mg G4: AREDS + L 10 mg + Z 2 mg + DHA 350 mg + EPA 650 mg	No effect in reducing progression to advanced AMD. No effect in improving VA. In the lowest quintile of L dietary intake, L + Z had significant effect vs. no L + Z in reducing progression to advanced AMD.
AREDS2 (2014) [105]	RCT, participants with bilateral drusen or AMD in 1 eye (3335 eyes analyzed); 3 groups: G1 (1114 eyes); G2 (1104 eyes); G3 (1117 eyes)	G1: AREDS + L 10 mg + Z 2 mg without beta-carotene; G2: AREDS + L 10 mg + Z 2 mg; G3: AREDS formulation	G1 (compared to G3) significantly reduced progression to advanced AMD and neovascular AMD, no effect for CGA. No difference between G2 vs. G3.
Akuffo et al. (2015) [92]	Intervention trial, participants with AMD (67); 3 groups with different dosages	G1: L 20 mg + Z 2 mg; G2: L 10 mg + Z 2 mg + MZ 10 mg; G3: L 3 mg + Z 2 mg + MZ 17 mg	After 3 years, all the groups showed a significant increase in MPOD but no effects in reducing progression to advanced AMD or improving VA. CS significantly increased, mainly in G3.
Beatty et al. (2013) [15]	RCT, participants with at least bilateral early AMD (433); intervention group (216) vs. placebo (217)	Intervention group: formulation containing L 12 mg	No significant improvement in CS. Significant VA enhancement not before 24 months.
Berrow et al. (2013) [26]	RCT, participants with AMD (14); treatment group (8) vs. no treatment (6)	Treatment group: L 12 mg	After 40 weeks, no clinical effects; only minimal improvement in mfEGR.
Bone (2010) [38]	Intervention trial, healthy participants (87); 4 groups: G1 (10); G2 (17); G3 (22); G4 (38)	G1: placebo; G2: L 5 mg;G3: L 10 mg; G4: 20 mg	MPOD increased in a linear, dose-dependent manner. L did not increase MPOD in all the participants.
Cangemi (2007) [101]	Intervention trial, participants with at least 1 eye with dry AMD (37)	Formulation containing L 8 mg	Significant improvement in VA after 6 months.
Dawczynski et al. (2013) [35]	RCT, participants with non-exudative AMD (145); 3 groups: G1 (50); G2 (55); G3 (40)	G1: L 10 mg + Z 1 mg + DHA 100 mg + EPA 30 mg; G2: L 20 mg + Z 2 mg + DHA 200 mg + EPA 60 mg; G3: placebo	Significant increase in MPOD and improvement in VA both in G1 and G2; MPOD decreased in G3. No significant differences in MPOD accumulation between G1 and G2.
Fujimura et al. (2016) [96]	Intervention trial, participants with at least 1 eye with wet AMD or early AMD (20)	Formulation containing L 20 mg + Z 1 mg + DHA 200 mg	After 6 months, significant increase in MPOD and CS. Linear correlation between MPOD and serum concentrations of L.
Hammond et al. (2014) [97]	RCT, healthy participants (115); intervention group (58) vs. placebo (57)	Intervention group: formulation containing L 10 mg + Z 2 mg	After 1 year, significant increase in MPOD, recovery from photostress and chromatic contrast.
Huang et al. (2015) [94]	RCT, participants with early AMD (108); 4 groups: G1 (28); G2 (26); G3 (27); G4 (27)	G1: placebo; G2: L 10 mg; G3: L 20 mg; G4: L 10 mg + Z 10 mg	After 2 years, significant increase in MPOD and mean retinal sensitivity.
Huang et al. (2015) [36]	RCT, participants with early AMD (108); 4 groups: G1 (28)]; G2 (26); G3 (27); G4 (27)	G1: placebo; G2: L 10 mg; G3: L 20 mg; G4: L 10 mg + Z 10 mg	After 2 years, significant increase in MPOD and CS, no effect in VA and flash recovery time. Same efficacy in all treatment groups.
Ma et al. (2009) [23]	Intervention trial, healthy participants (37); 3 groups: G1 (12); G2 (12); G3 (13)	G1: placebo; G2: L 6 mg; G3: L 12 mg	After 12 weeks, no effect in improving VA and glare sensitivity. CS significantly increased in both G2 and G3, but much more in G3.
Ma et al. (2012) [37]	RCT, participants with early AMD (108); 4 groups: G1 (27); G2 (27); G3 (27); G4 (27); group of healthy controls (36)	G1: placebo; G2: L 10 mg; G3: L 20 mg; G4: L 10 mg + Z 10 mg	After 48 weeks, both G3 and G4 effectively increased MPOD; CS only improved on G3. Positive correlation between MPOD increase, VA and CS. Significant dose-response effect following L supplementation.
Murray et al. (2013) [16]	RCT, participants with early AMD (72); intervention group (36) vs. placebo (36)	Intervention group: formulation containing L 10 mg	Significant effect on MPOD. No improvement in VA, but VA decreased on placebo. Changes in VA were significant between L and placebo.
Nolan et al. (2011) [98]	RCT, healthy participants (121); intervention group (61) vs. placebo (60)	Intervention group: formulation containing L 12 mg + Z 1 mg	After 1 year, significant effect on MPOD but no improvement in VA, CS, glare disability, recovery from photostress.
Obana et al. (2015) [25]	RCT, healthy participants (36)	L 10 mg + Z 1 mg	After 6 months, no effect on MPOD. Only a subgroup of 13 participants had an effective increase both in serum levels of L and MPOD.
Parisi et al. (2008) [13]	RCT, participants with non-advanced AMD (27); treatment group (15) vs. no treatment [12]	Treatment group: formulation containing L 10 mg + Z 1 mg	After 1 year, significant improvement in central retina dysfunction but no effect in peripheral retina.
Piermarocchi et al. (2012) [102]	Intervention trial, participants with dry AMD [109]; treatment group [84] vs. no treatment [26]	Treatment group: formulation containing L 10 mg + Z 1 mg	Significant improvement in VA and CS after 2 years.
Richer et al. (2004) [12]	RCT, participants with atrophic AMD (90); 3 groups: G1 (29); G2 (30); G3 (31)	G1: L 10 mg; G2: formulation containing L 10 mg; G3: placebo	After 1 year, both G1 and G2 showed significantly increased MPOD, VA and CS.
Richer et al. (2011) [95]	RCT, participants with non-advanced AMD (60); 3 groups: G1 (10); G2 (25); G3 (25)	G1: L 9 mg; G2: Z 8 mg; G3: L 9 mg + Z 8 mg	After 1 year, both G1 and G2 showed effectively increased MPOD and CS; no improvement in G3.
Rosenthal et al. (2006) [22]	Intervention trial, participants with AMD (45); 3 groups: G1 (15); G2 (15)]; G3 (15)	G1: L 2.5 mg; G2: L 5 mg; G3: L 10 mg	After 6 months, no effect in VA. 10 mg were safely administered without toxicity or adverse events.
Sabour-Pickett et al. (2014) [100]	Intervention trial, participants with AMD (52); 3 groups: G1 (17); G2 (21); G3 (14)	G1: L 20 mg + Z 2 mg; G2: L 10 mg + Z 2 mg + MZ 10 mg; G3: L 3 mg + Z 2 mg + MZ 17 mg	After 1 year, MPOD increased in all groups; the significant improvement in CS was much more effective in G3.
Sasamoto et al. (2011) [24]	Intervention trial, healthy controls (5), participants with AMD (33) and participants with central serous chorioretinopathy (5)	Formulation containing L 6 mg	After 1 year, no effect in MPOD. Significant improvement in CS and retinal sensitivity.
Stringham et al. (2016) [34]	RCT, healthy participants (28); 4 groups: G1 (5); G2 (7); G3 (8); G4 (8)	G1: placebo; G2: L 6 mg + Z 0,7 mg + MZ 0,5 mg; G3: L 10.9 mg+Z 1.3 mg + MZ 0.9 mg; G4: L 22 mg + Z 2.7 mg + MZ 2 mg	All the intervention groups showed a significant effect in MPOD at 12 weeks, G3 was much more effective.
Weigert et al. (2011) [14]	RCT, participants with AMD (126); 2 groups: G1 (84); G2 (42)	G1: L 20 mg for the first 3 months, L 10 mg for the last 3 ones; G2: placebo	After 6 months, MPOD increased by nearly 28% vs. placebo in G1. No improvement in VA and macular function. The lower MPOD at baseline, the greater the improvement. Linear correlation between changes in MPOD, VA and macular function.
Wolf-Schnurrbusch et al. (2015) [99]	Intervention trial, participants with AMD (79); 2 groups: G1 (40); G2 (39)	G1: formulation containing L 10 mg + Z 1 mg; G2: formulation containing L 10 mg + Z 1 mg + DHA and EPA 130 mg	After 6 months and 1 year, MPOD and CS [not VA] significantly increased G1. No significant effect on G2.
Yao et al. (2013) [93]	RCT, healthy participants (120); treatment group (60) vs. placebo (60)	Treatment group: L 20 mg	After 1 year, significant improvement in MPOD, CS and glare sensitivity vs. placebo. No effect in VA.

AMD: age-related macular degeneration; AREDS: age-related eye disease study (formulation: vit. C: 500 mg; vit. E: 400 UI; beta carotene: 15 mg; zinc: 80 mg; copper: 2 mg); CS: contrast sensitivity; DHA: docosahexaenoic acid; EPA: eicosapentaenoic acid; L: lutein; mfEGR: multifocal electroretinogram; MPOD: macular pigment optical density; MZ: meso-zeaxanthin; RCT: randomized controlled trial; VA: visual acuity; Z: zeaxanthin.
